# Fungal chemical warfare: the role of aflatoxin and fumonisin in governing the interaction between the maize pathogens, *Aspergillus flavus* and *Fusarium verticillioides*

**DOI:** 10.3389/fcimb.2024.1513134

**Published:** 2025-01-03

**Authors:** Timothy R. Satterlee, Jaci A. Hawkins, Trevor R. Mitchell, Qijian Wei, Jessica M. Lohmar, Anthony E. Glenn, Scott E. Gold

**Affiliations:** ^1^ United States National Poultry Research Center, United States Department of Agriculture Toxicology and Mycotoxin Research Unit, Athens, GA, United States; ^2^ Agricultural Research Service, United States Department of Agriculture, Food and Feed Safety Research Unit, New Orleans, LA, United States

**Keywords:** mycotoxins, *Aspergillus flavus*, *Fusarium verticillioides*, aflatoxin, fumonisin

## Abstract

The mycotoxigenic fungi, *Aspergillus flavus* and *Fusarium verticillioides*, commonly co-colonize maize in the field, yet their direct interactions at the chemical communication level have not been well characterized. Here, we examined if and how the two most infamous mycotoxins produced by these species, aflatoxin and fumonisin, respectively, govern interspecies growth and mycotoxin production. We showed that fumonisin producing strains of *F. verticillioides* suppressed the growth of *A. flavus* while non-producers did not. Additionally, while aflatoxin did not inhibit *F. verticillioides* growth, it did suppress fumonisin production. Fumonisin B_1_ concentration levels plummeted when challenged with a high dose of aflatoxin B_1_ or with an aflatoxin producing strain. With these findings, expression of the genetic regulators of secondary metabolism was investigated for both fungi. While no strong effect was seen on genes in the aflatoxin biosynthetic gene cluster when exposed to fumonisin B_1_, the fumonisin repressor *FvZBD1*, which is adjacent to the cluster, was induced with expression proportionate to concentration when *F. verticillioides* was challenged with aflatoxin B_1_. We also assessed the expression of the global regulators of fungal secondary metabolism, *veA* and *laeA*, and found that their expression is altered in both *A. flavus* and *F. verticillioides* when exposed to their competitor’s mycotoxin. This work gives insight into the ecological roles of mycotoxins and why these fungi may produce them as weapons in the interspecies battle for resource acquisition.

## 1 Introduction

Fungi produce secondary metabolites that enhance their environmental fitness, but these compounds are by definition not required for axenic survival (Avalos and Limón, 2022). The most famous fungal secondary metabolite is the antibiotic penicillin. Used in medicine to treat bacterial infections, this metabolite in nature allows its producer to gain a competitive advantage over bacteria by blocking their cell wall biosynthesis and thus suppressing their growth (Yocum et al., 1980). Mycotoxins are a well-studied subgroup of fungal secondary metabolites due to their toxic effects on humans and animals (Bennett and Klich, 2003). Additionally, the United Nations Food and Agriculture Organization has estimated that, in the United States and Canada alone, mycotoxins cause losses of up to $5 billion U.S. annually (Eskola et al., 2020).

Two infamous mycotoxins that are frequent contaminants in some agricultural products are aflatoxins and fumonisins. Aflatoxin was the first mycotoxin identified. It was associated with the Turkey X disease outbreak in the United Kingdom in 1960 where 100,000 poults were killed upon consumption of contaminated peanut meal (Pickova et al., 2021). Aflatoxins are primarily produced by *Aspergillus flavus* and *Aspergillus parasiticus* and were shown to be the most carcinogenic naturally occurring compounds known with demonstrated toxicity in the parts per billion range (Trucksess and Diaz-Amigo, 2011). Currently, aflatoxin is under strict regulation in the United States and European Union to limit how much enters the food chain targeted for human consumption (20 parts per billion (ppb)) and animal feed (50-100 ppb for poultry). Fumonisin, a mycotoxin primarily produced by *Fusarium* spp., has also been associated with a plethora of health problems in animals. Currently, there are only recommended guidelines for the permissible amount of fumonisin in food and feed, with the standard for poultry broiler feed set at 100 parts per million (ppm) (Alshannaq and Yu, 2017).

In addition to addressing the toxicity of mycotoxins to animals, several studies have examined other roles these compounds may have that could provide a selective advantage to the producing fungi. When confronted with oxidative stress, *A. flavus* increases production of aflatoxin (Fountain et al., 2016b, a). Strains of *A. flavus* that produced more aflatoxin have demonstrated increased resistance to oxidative stress (Fountain et al., 2015). It has been hypothesized that aflatoxin acts as an antioxidant to scavenge free radicals, thus protecting the fungus from the oxidative stress it typically encounters upon colonizing plants (Finotti et al., 2021). In *F. verticillioides*, there is evidence that fumonisin can be a virulence factor aiding in infection and symptom development in susceptible lines of corn (Glenn et al., 2008; Blacutt et al., 2018; Gao et al., 2020). Fumonisin B1 has been reported to have antifungal activity against *Alternaria alternata, Pencillium expansum*, and *Fusarium graminearum*, the later being a competing pathogen in corn in some regions (Keyser et al., 1999).

Both *A. flavus* and *F. verticillioides* are common colonizers of corn, infecting and contaminating the crop with their harmful mycotoxins. Numerous studies have found both fungal species occupying the same field as recently reviewed by Chen et al (Chen et al., 2023). Several of these studies have also found both aflatoxin and fumonisin in the same kernels indicating that both fungi can colonize the same host simultaneously. Examination of the interactions between these two fungi revealed that direct competition impacts their growth and the production of their respective mycotoxins, but none have examined the direct effect the individual mycotoxins have in their competition (Shu et al., 2017; Chen et al., 2021; Lanubile et al., 2021). Based on the hypothesis that *A. flavus* and *F. verticillioides* encounter each other’s mycotoxins when competing to colonize corn kernels, we investigated the specific role of aflatoxin and fumonisin in this dual fungus competitive interaction. To achieve this, we performed direct competition assays with wild-type toxin producing strains compared to interactions with non-toxigenic mutant strains of *A. flavus* (Δ*aflR*) and *F. verticillioides (Δfvfum1).* The direct effect of each mycotoxin on its competitor was also assayed focusing on growth, mycotoxin production, and the expression of selected genes impacting secondary metabolism.

## 2 Materials and methods

### 2.1 Fungal strains used and growth conditions

Wild-type strains NRRL3357 and FRCM3125 (FGSC7600) were used for *A. flavus* and *F. verticillioides*, respectively. The aflatoxin non-producing strain, a Δ*aflR* (Δ*aflR::ptrA*) mutant in the NRRL3357 background was generated for this study, while the fumonisin non-producing strain was from a previous study that has a defective *FvFum1* (FVEG_00316) gene (derived from FRC M3125, *Δfvfum1::hyp)* (Proctor et al., 1999; Desjardins et al., 2002). Stocks of each strain were stored in 30% glycerol at -80°C. To maintain the stock and/or generate spores for inoculum, cultures were grown on Potato Dextrose Agar (PDA) (Neogen, Lasing, Michigan, USA) or double strength 5/2 agar (100 mL V8 juice, 40 g agar, pH 5.2 per liter of medium) unless specified differently (Chang et al., 1993).

### 2.2 Construction and confirmation of an *Aspergillus flavus* Δ*aflR* strain

To construct an *Aspergillus flavus* Δ*aflR* strain, 1.5 kb DNA fragments containing upstream and downstream sequences flanking the *aflR* (*F9C07_7811*) coding region were amplified by PCR from gDNA of the *A. flavus* NRRL3357 wild-type strain utilizing the primers P1/P2 and P3/P4, respectively. The 2.0 kb pyrithiamine resistance gene (*ptrA*) was PCR amplified from the commercial pPTRI vector (TaKaRa Bio Inc., Shiga, Japan) using primers P5/P6. Fusion PCR was carried out with the primers P7/P8 as previously described (Szewczyk et al., 2006) to generate a 4.8 kb *aflR* deletion cassett. Protoplast and CaCl_2_-PEG mediated fungal transformation of the *aflR* deletion cassette into the NRRL3357 wild-type strain was carried out as previously described (Chang, 2008). Transformants were selected on Czapek-dox (BD Difco, Franklin Lakes, NJ, USA) regeneration plates supplemented with 0.1 µg/L pyrithiamine hydrobromide (Sigma-Aldrich, Burlington, MS, USA). Colonies displaying resistance to pyrithiamine hydrobromide were subcultured. Fungal mycelium used for genomic DNA isolation was cultivated by inoculating 10^6^ spores/mL into 50 mL of PDB (PDB; EMD, Darmstadt, Germany) medium. The cultures were incubated at 250 rpm at 30°C for 24 h prior to harvesting mycelium and extracting genomic DNA using a Zymo Quick-DNA Fungal/Bacterial Miniprep Kit (Zymo Research, Irvine, CA, USA). Diagnostic PCRs were carried out by using either OneTaq 2X Master Mix (New England BioLabs, Ipswich, MA, USA) or Phire PCR Master Mix (Thermo Scientific, Watham, MA, USA) with location-specific primers to confirm the knockout mutants of *aflR* gene. Thermocycler settings used were set according to manufactures recommendations. All primers utilized in creating this strain are listed in **Supplementary Table S1**.

### 2.3 Direct confrontation assays

Using standard 100 mm disposable Petri plates with 25 ml of PDA, *A. flavus* and *F. verticillioides* strains were point inoculated 40 mm apart and equidistant from the center of the plate. 5 µl of a spore suspension (10^6^ spores/ml) was used for plate spot inoculations. Plates were incubated for up to seven days in the dark at 28°C. For mycotoxin quantification, cultures were grown for five days, which allowed for sufficient growth without colonies making direct contact. After incubation cultures were photographed and cores were taken if required for mycotoxin analysis. For each pairing, five 5 mm diameter plugs were taken with a sterile cork borer from five locations across the plate: from the center in between colonies, 5 mm from the colony edge closest (proximal) to the center of the plate, and 5 mm from the colony edge farthest (distal) from the center. Proximal and distal sample cores were collected from both fungi on each plate. Controls were monocultures of the wild-type *A. flavus* or *F. verticillioides*. Cores from the edge of the wild-type colonies were used for comparison to both the distal and proximal plugs in the dual interactions. All five cores from each sample were submerged in 10 ml of 50% acetonitrile (+ 5% formic acid) and extracted overnight. Samples were then diluted to a total concentration of 30% acetonitrile before being analyzed for aflatoxin and fumonisin by Liquid Chromatography Mass Spectrometry (LC/MS).

### 2.4 Mycotoxin exposure assays

Stocks of both aflatoxin B_1_ and fumonisin B_1_ were purchased from Cayman Chemical (Niles, Illinois, USA). Aflatoxin stocks were created using a 50:50 mixture of acetone:methanol while fumonisin was dissolved in sterile water. Various concentrations of each mycotoxin, up to 100 µg/ml, were added to 1 ml molten PDA in 24-well plates. 5 µl of a spore suspension (10^6^ spores/ml) from the wild-type strain of *A. flavus* or *F. verticillioides* was placed in each well. Cultures were grown for 72 h, photographed, and the entire contents of each well were harvested for quantification of aflatoxin or fumonisin content by LC/MS as stated above.

### 2.5 Gene expression analysis of response to mycotoxins

To measure the response of each fungus to its competitor’s mycotoxin, wild-type strains of either *A. flavus* or *F. verticillioides* were inoculated into 3 ml of Potato Dextrose Broth (PDB, Neogen) at a final concentration of 10^5^ spores/ml. At the time of inoculation, aflatoxin B_1_ was added to *F. verticillioides* cultures and fumonisin B_1_ was added to *A. flavus* cultures both at a concentration of 20 µg/ml. Cultures were grown at 28°C in the dark and shaken at 250 rpm for up to 96 h. At 72 h and 96 h, cultures were destructively sampled with mycelia separated from culture supernatants. *A. flavus* mycelium was collected by filtering culture through sterile Miracloth (Millipore Sigma). 1.5 ml of *F. verticillioides* cultures were collected into prechilled 1.7 ml microcentrifuge tubes and then pelleted at 8000 rpm at 4°C. Collected mycelia was then flash frozen in liquid nitrogen and immediately stored at -80°C until ready for RNA extraction. Additional separate biological replicates (mycelia and supernatant) were collected for analysis of aflatoxin and fumonisin production. Equal volumes of 100% acetonitrile (+ 5% formic acid) were added to the cultures and extracted overnight. Extracts were diluted to 30% acetonitrile with water and analyzed via LC/MS.

For extracting RNA from *F. verticillioides* mycelia, the PureLink RNA Mini Kit (Invitrogen, Waltham, MA, USA) was used following manufacturer instructions. Homogenization was performed using a MP Biomedical (Santa Ana, CA, USA) FastPrep with Lysing Matrix D tubes. For *A. flavus*, RNA extraction was done using the manufacturer protocol for a TRIzol extraction with cleanup done using the RNeasy Mini Kit (Qiagen). RNA quantity and quality was determined using an Agilent 2100 Bioanalyzer with RIN values above 6 acceptable for further processing. RNA was treated with RQ1 DNAse (Promega, Madison, WI, USA) following the manufacturer protocol. To create cDNA, a Moloney murine leukemia virus (MMLV) (Promega) reverse transcriptase was used on the DNAse treated RNA. qRT-PCR was performed on a Bio-Rad CFX96 Real-Time System using SYBR green dye for fluorescence detection. Gene expression was normalized to expression of the 18S ribosomal RNA gene for *A. flavus* and the β-Tubulin gene (FVEG_04081) for *F. verticillioides.* Expression was assessed by the 2^-ΔΔCT^ method (Sherif et al., 2023). All primer sequences used in qRT-PCR analysis are listed in **Supplementary Table S2**.

### 2.6 Statistical analysis

All statistical analyses were done using R version 4.4.1. An analysis of variance (ANOVA) was performed on data generated in each experiment alongside a Tukey multiple-comparison test. To determine significance between treatments a *P* value of <0.05 was set.

## 3 Results

### 3.1 *Fusarium verticillioides* inhibits *Aspergillus flavus* growth through fumonisin

**Figure 1:**
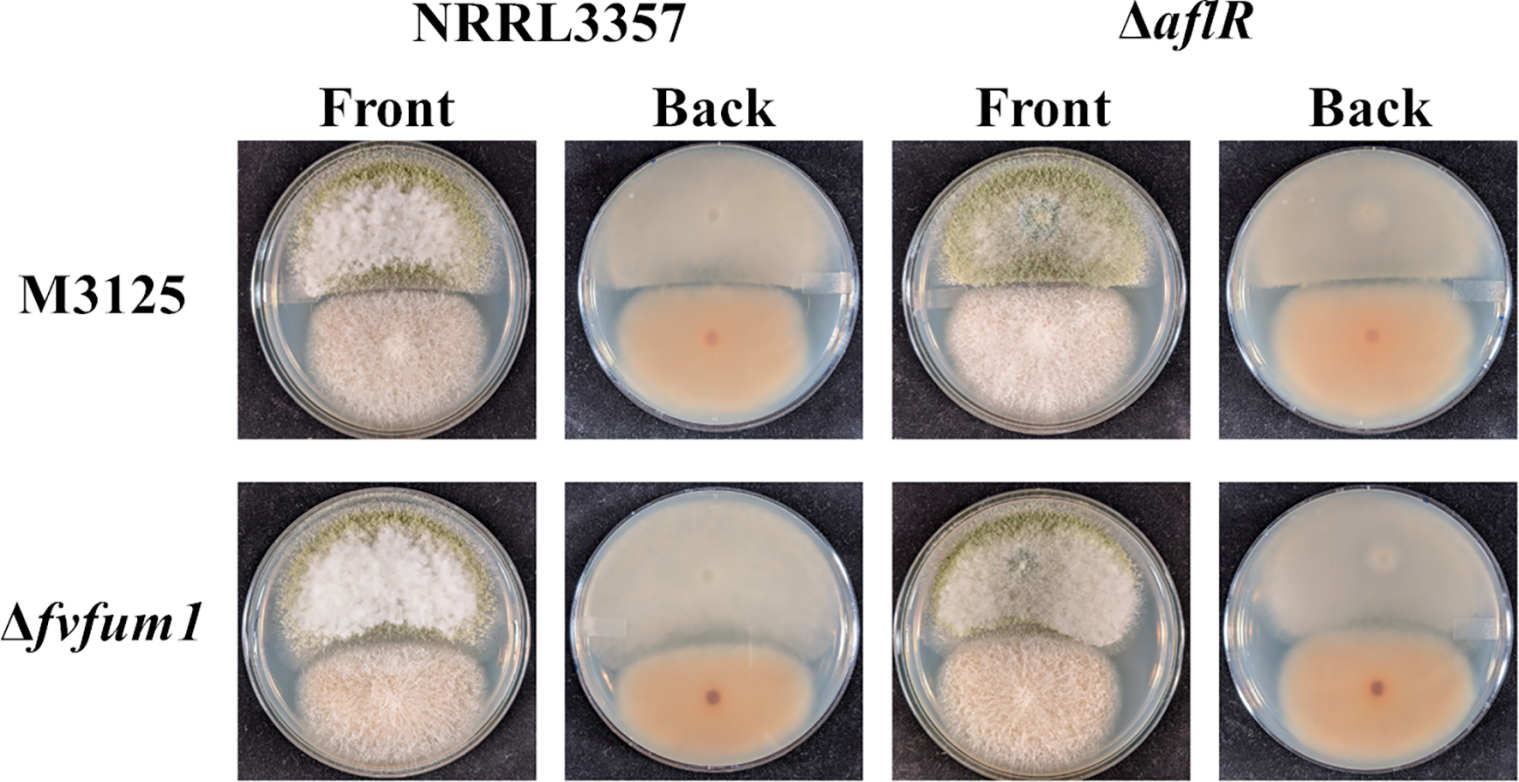
Fumonisin plays a role in *F. verticillioides* inhibition of *A. flavus* growth. Wild-type and/or atoxigenic mutant (Δ*aflR* and/or Δ*fvfum1*) strains of both *A. flavus* (*Af*) and *F. verticillioides* (*Fv*) were point inoculated onto PDA and grown for 7 days at 28°C in the dark. Experiment was done three different times with three biological replicates.

Initially, wild-type strains of *A. flavus* and *F. verticillioides* were confronted with each other on PDA. Inhibition of *A. flavus* growth was observed as a sharp line of demarcation, beyond which it did not grow (**Figure 1**). To determine if aflatoxin and/or fumonisin were involved in this interaction, nonproducing mutants were utilized. For this study, an aflatoxin non-producing *aflR* deletion strain was created as described in the Methods section above (**Supplementary Figure S1A**). Pyrithiamine resistant transformants were further confirmed with diagnostic PCR (**Supplementary Figures S1B–E**). For a fumonisin non-producing strain of *F. verticillioides*, a strain with a disruption of the *FvFUM1* gene from previous work was utilized (Proctor et al., 1999; Desjardins et al., 2002). The aflatoxin non-producing mutant (Δ*aflR*) confronted with wild-type *F. verticillioides* showed an even sharper demarcation line than observed with wild type *A. flavus*, suggesting that aflatoxin diminishes the competitive edge of *F. verticillioides* over *A. flavus* (**Figure 1**). Likewise, when confronted with the fumonisin non-producing mutant (Δ*fvfum1*) the growth of wild-type *A. flavus* was clearly less inhibited, and its colony made direct contact with the fumonisin non-producing mutant. Additionally, when non-toxigenic competitor strains are confronted, the result is similar to the *A. flavus* wild-type vs Δ*fvfum1* interaction with the colonies making contact. These results indicate that both aflatoxin and fumonisin are, in part, responsible for competitor growth inhibition in these interactions.

### 3.2 Aflatoxin suppresses production of fumonisin in *F. verticillioides*

**Figure 2:**
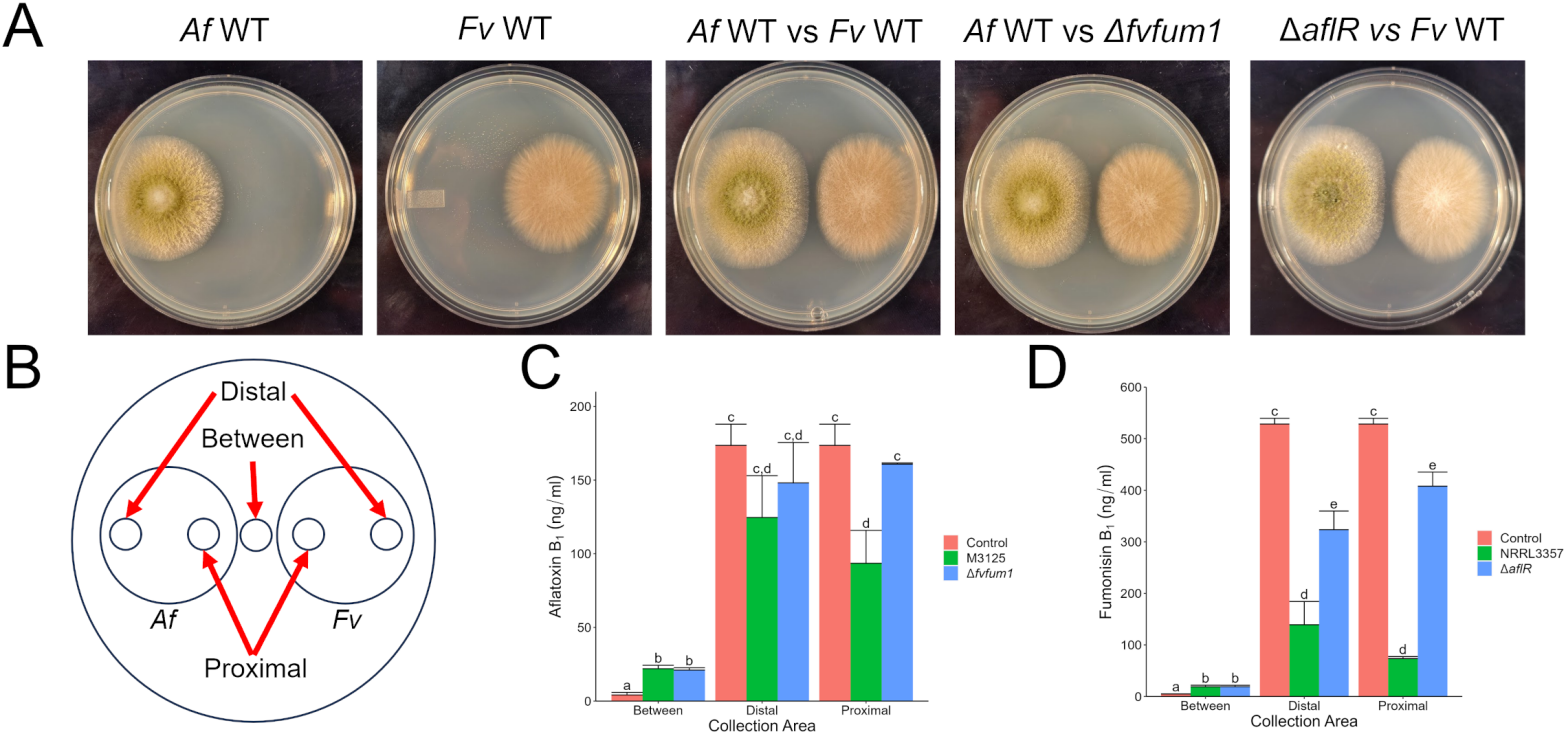
In direct competition with *A. flavus*, aflatoxin suppresses fumonisin production by *F*. *verticillioides.* **(A)** *A*. *flavus (Af)* and *F*. *verticillioides* (*Fv*) strains grown for 5 days at 28°C in the dark. **(B)** Collection sites from fungal cultures for mycotoxin analysis via LC/MS. **(C)** Aflatoxin B_1_ production by *A*. *flavus* in response to *F*. *verticillioides* wild-type and Δ*fvfum1* strains. **(D)** Fumonisin B_1_ analysis of *F*. *verticillioides* culture in response to *A*. *flavus* wild-type and Δ*aflR* strains. The controls used in C & D were the monocultures shown in **(A)** Different letters above each column indicate that the values are statistically different (P<0.05) based on results of an ANOVA run with Tukey test comparison. Experiment was three done different times with three biological replicates.

With the demonstrated effect of mycotoxin production on their competitor’s growth, we investigated the effect each mycotoxin had on its competitor’s mycotoxin production. Using the same strains as above, *F. verticillioides* and *A. flavus* were point inoculated equidistant from each the plate center and grown for 5 days to prevent direct contact (**Figure 2A**). Agar plugs were collected from three locations: between colonies, the proximal edge of each colony closest to its competitor, and the distal edge (**Figure 2B**). This was done to determine if the effects of mycotoxin exposure were localized to one area or if a colony-wide response was caused. In response to the competitor fungus, both *A. flavus* and *F. verticillioides* secreted mycotoxins at higher levels than monoculture controls (**Figures 2C, D**).

Compared to monoculture controls, when *A. flavus* encountered a wild-type strain of *F. verticillioides*, aflatoxin production was reduced proximally as compared to the other treatments. No statistical differences were found at the distal colony edge (**Figure 2C**). After exposure to aflatoxin producing strains, fumonisin production by *F. verticillioides* was suppressed at both proximal and distal collection sites (**Figure 2D**). At distal collection sites, no aflatoxin was detected (data not shown) indicating the response is not a localized effect but is rather colony-wide. Additionally, the Δ*aflR* strain also caused slight inhibition of fumonisin production, suggesting other metabolites produced in these interspecies interactions likely play roles.

### 3.3 Antagonistic mycotoxins directly influence gene expression of competitor regulators of secondary metabolism

**Figure 3:**
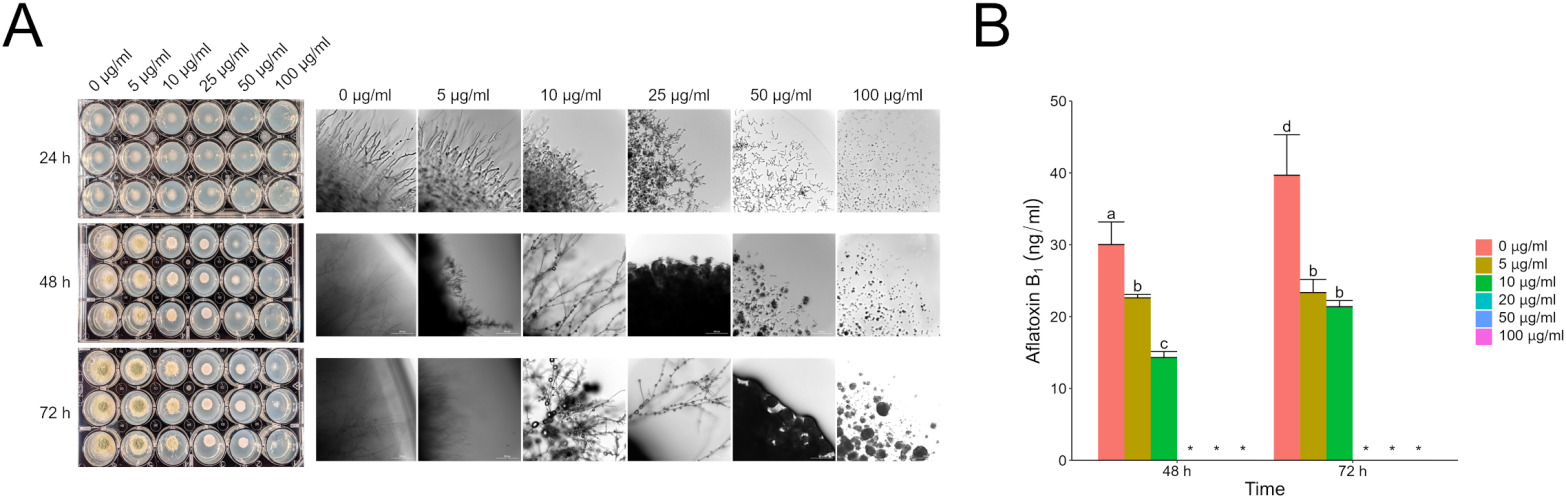
Direct effect of fumonisin B_1_ on *A. flavus* growth and aflatoxin B_1_ production. **(A)** *A*. *flavus* culture treated with increasing concentrations of FB_1_ as shown in **(B)**. Cultures were grown in 24-well plates for 72 hours at 28°C in the dark with micrographs taken every 24 hours. **(B)** LC/MS analysis of AFB_1_ content in culture at 48 and 72 hours. Different letters above each column indicate that the values are statistically different (P<0.05) based on results of an ANOVA run with Tukey test comparison. In panel B, “*” indicates samples that were not detected or quantifiable by LC/MS. Experiment was done three different times with three biological replicates.

**Figure 4:**
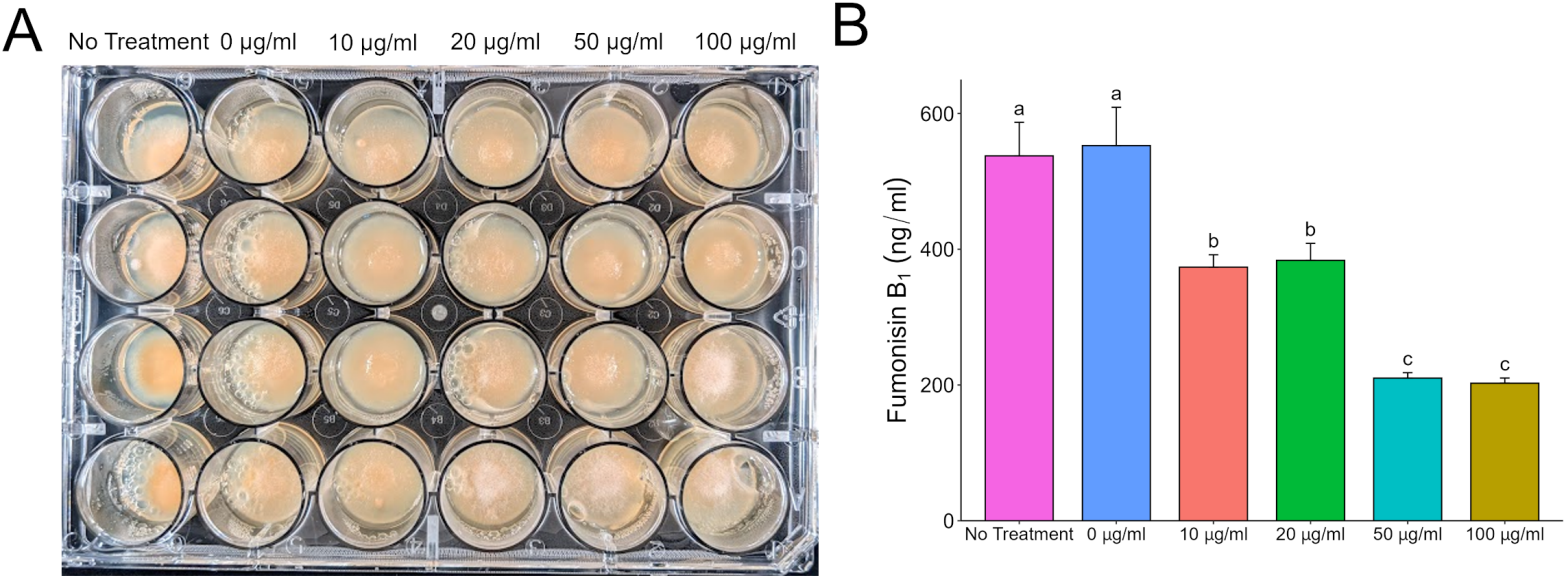
Direct effect of aflatoxin B_1_ on *F*. *verticillioides* growth and fumonisin B_1_ production. **(A)** *F*. *verticillioides* treated with increasing concentrations of AFB_1_ up to 100 µg/ml and grown in 24-well plates for 72 hours at 28°C in the dark. Samples labeled as “0 µg/ml” are samples that have a volume of 1% acetone:methanol (50:50) with no aflatoxin added to represent the carrier used to administer aflatoxin to the *F*. *verticilliodes* as a 0 µg/ml treatment. The “No Treatment” sample do not have the acetone:methanol carrier added to the samples. **(B)** LC/MS analysis of FB_1_ content in culture at 72 hours. Different letters above each column indicate that the values are statistically different (P<0.05) based on results of an ANOVA run with Tukey test comparison. Experiment was done three different times with three biological replicates.

Filamentous fungi like *Aspergillus* and *Fusarium* are known to produce a plethora of secondary metabolites. Some of these metabolites have been characterized, but there are apparently more that have yet to be identified. To focus our efforts, we decided to evaluate the direct effects of purified aflatoxin B_1_ (AFB_1_) and fumonisin B_1_ (FB_1_) on *F. verticillioides* and *A. flavus*, respectively. Both mycotoxins are commercially available in relatively pure form (>98%) and are also the primary forms of the respective toxins. Testing FB_1_ on *A. flavus* demonstrated the same phenotypes as observed with the wild type fumonisin producer, with FB_1_ inhibiting *A. flavus* growth and aflatoxin production. At 24 h with a 100 µg/ml dose of FB_1_, the *A. flavus* spores were yet to germinate; at 72 h the cultures had active vegetative growth. This indicates that FB_1_ was fungistatic providing a head start but not fungicidal towards *A. flavus* at concentrations up to 100 µg/ml (**Figure 3**). The original phenotype of fumonisin suppression when exposed to wild type *A. flavus* also held true with large amounts (up to 100 µg/ml) of AFB_1_ suppressing fumonisin production but having little to no effect on *F. verticillioides* colony growth (**Figure 4**).

**Figure 5:**
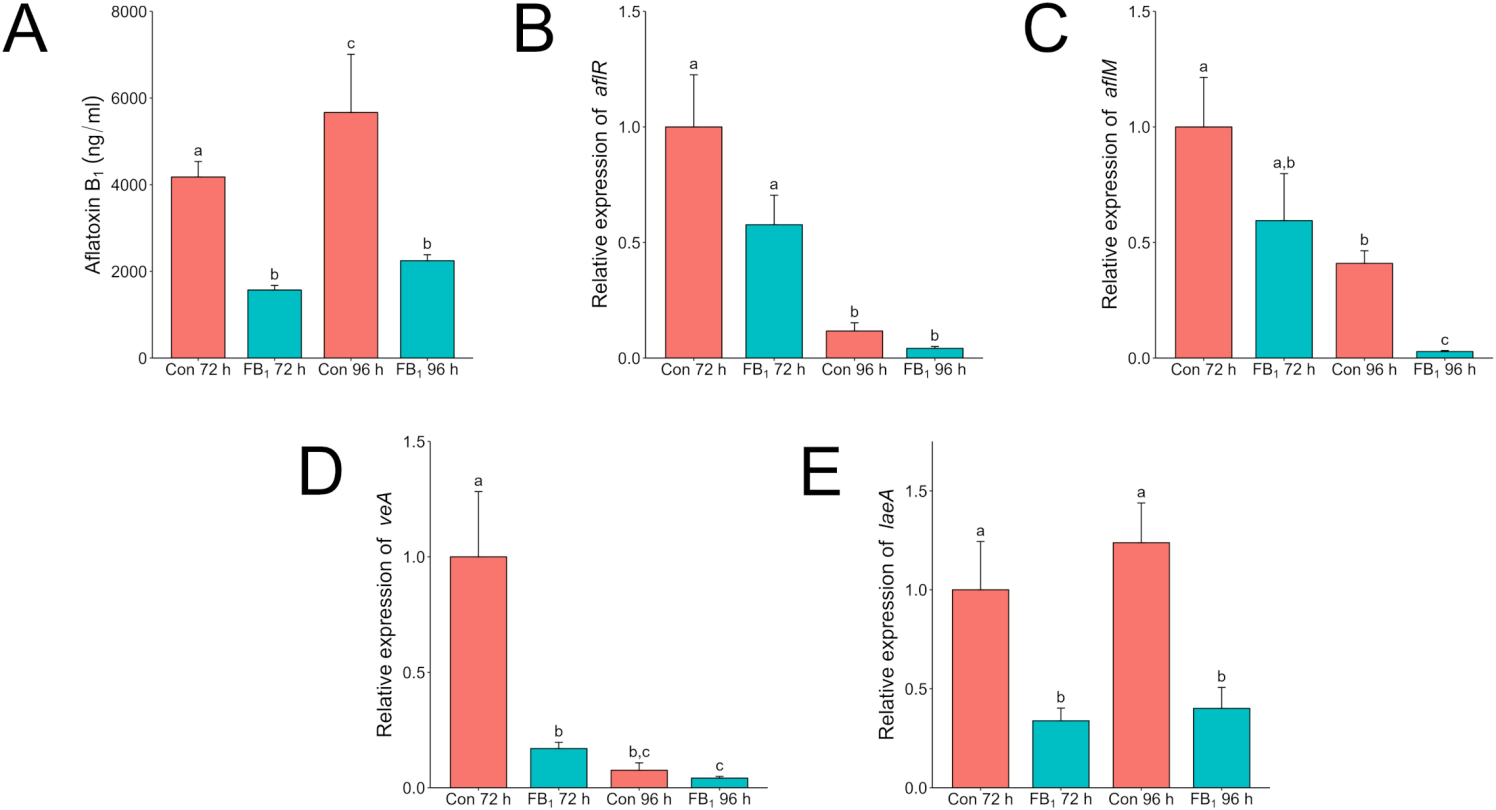
Fumonisin B_1_ impacts aflatoxin production and the expression of velvet regulators *veA* and *laeA* in *A*. *flavus* in liquid culture*. A. flavus* wild type was grown in 3 ml of PDB with or without 20 µg/ml fumonisin B_1_ for 72 or 96 h. The “Con” or control treatment are cultures that do not have any fumonisin added and “FB_1_” are cultures with 20 µg/ml of fumonisin B_1_ added. **(A)** Complete samples, mycelia and supernatant, were collected at 72 and 96 h and analyzed via LC/MS for aflatoxin B_1_ content. Mycelia were also collected from additional tubes for RNA extraction. Relative expression of *aflR* **(B)**, *aflM* **(C)**, veA **(D)**, and laeA **(E)** was analyzed by qRT-PCR. Expression values were normalized to the 72-hour control. Different letters above columns indicate that the values are statistically different (P<0.05) based on results of an ANOVA run with Tukey test comparison. Experiment was done three different times with four biological replicates.

After validating the effect of the individual mycotoxins, we assessed the expression of selected mycotoxin regulatory and biosynthetic genes using subinhibitory doses of each against its appropriate competitor. Mycotoxins were administered at the time of inoculation. Liquid cultures were grown for up to 96 h, with separate full cultures (mycelia and supernatant) destructively sampled at 72 and 96 h for both RNA extraction and LC/MS analysis. Shaken *A. flavus* broth cultures exposed to fumonisin had significant reduction in aflatoxin production compared to control cultures without added fumonisin (**Figure 5A**). Surprisingly, no significant statistical differences in expression of *aflR*, which encodes the key activating transcription factor in the aflatoxin biosynthetic gene cluster, was found. However, there was a significant decrease in expression of *aflM* at 96 h (**Figures 5B, C**). The aflatoxin biosynthetic cluster gene, *aflM (*previously known as *ver-1*), encodes versicolorin dehydrogenase and has been effectively used to show activation of the aflatoxin biosynthetic pathway (Calvo et al., 2004; Raruang et al., 2020). The decrease in expression was not temporally correlated with a decrease in aflatoxin. Outside the biosynthetic cluster, two global regulators of morphological development and secondary metabolism, *veA* and *laeA*, were also assessed (**Figures 5D, E**) (Calvo et al., 2016). In *A. flavus* samples exposed to fumonisin B_1_ expression of *veA* was suppressed at 72 h, while expression in the control reached the same level 24 h later at the 96 h timepoint. The gene *laeA* was more consistent in expression that in the fumonisin treated samples it showed a significant decrease in expression at both time points.

**Figure 6:**
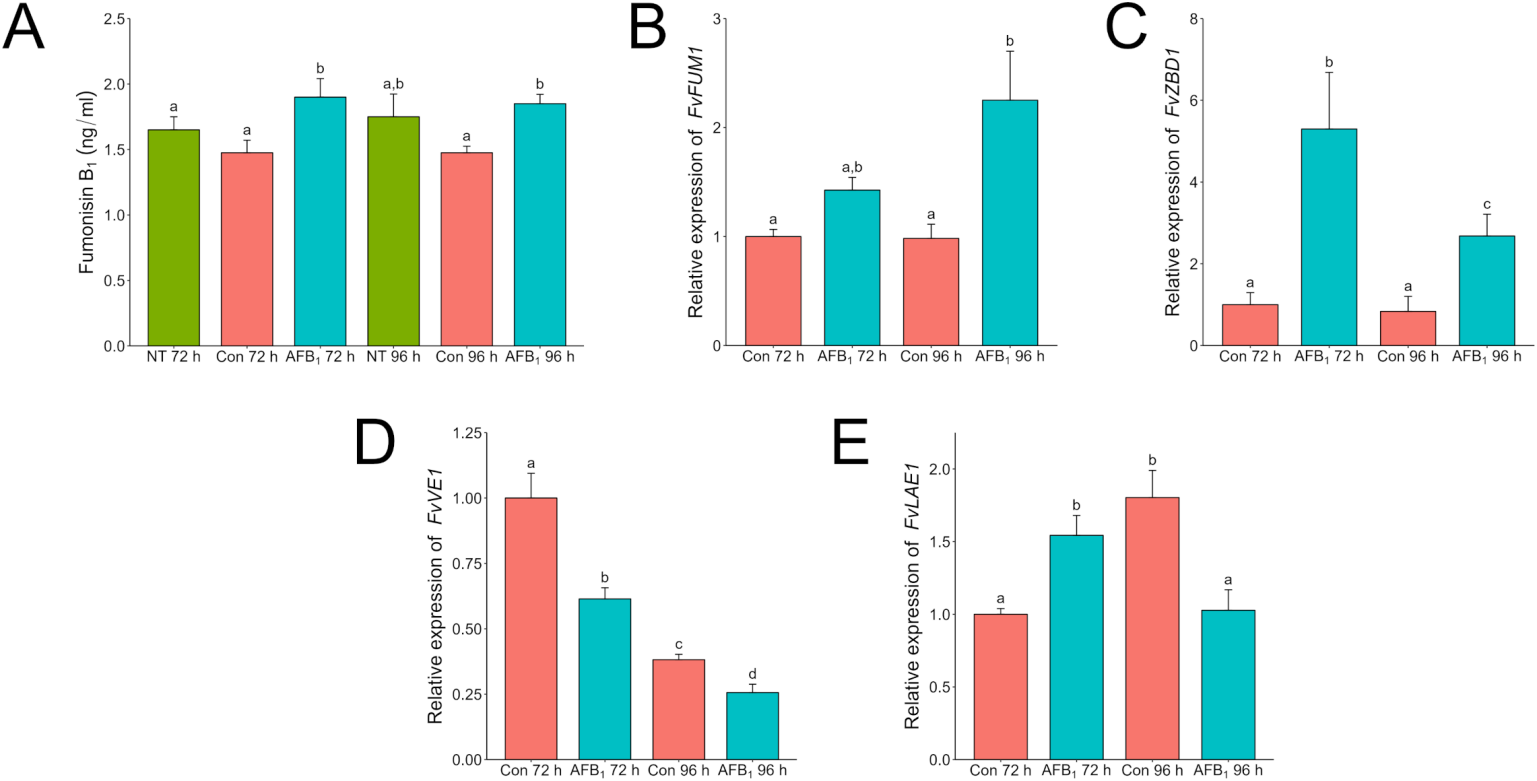
Aflatoxin B_1_ influences Fumonisin B_1_ production likely through induction of *FvZBD1* in *F*. *verticillioides. F. verticillioides* wild-type was grown in 3 ml of PDB with or without 20 µg/ml Aflatoxin B_1_ for 96 h. Samples labeled as “Con” are samples that have a volume of 1% acetone:methanol (50:50) with no aflatoxin added to represent the carrier used to administer aflatoxin to the *F*. *verticilliodes* as a 0 µg/ml treatment. The “No Treatment” sample do not have the acetone:methanol carrier added to the samples. Labels with “AFB_1_” are cultures with 20 µg/ml of aflatoxin B_1_ added. **(A)** Complete samples, mycelia and supernatant, were collected at 72 and 96 h and analyzed via LC/MS for aflatoxin B_1_. Mycelia from duplicate tubes were collected for RNA extraction. “NT” is a no treatment control. Samples labeled as “Control” are samples that have a volume of 1% acetone:methanol (50:50) with no aflatoxin added to represent the carrier used to administer aflatoxin to the *F*. *verticilliodes* as a 0 µg/ml treatment. Relative expression of *FvFUM1* **(B)**, *FvZBD1* **(C)**, *FvVE1* **(D)**, and *FvLAE1* **(E)** was analyzed by qRT-PCR. Expression values were normalized to the 72-hour control. Different letters above each column indicate that the values are statistically different (P<0.05) based on results of an ANOVA run with Tukey test comparison. Experiment was done three different times with four biological replicates.

In contrast to results on solid medium, *F. verticillioides* liquid shaken cultures produced more fumonisin at 72 and 96 h when dosed with aflatoxin than when not (**Figure 6A**). In response to aflatoxin treatment expression of the fumonisin biosynthetic gene cluster, a polyketide synthase encoding gene (*FvFUM1* (FVEG_00316)), increased over time while the gene for zinc-binding dehydrogenase (*FvZBD1)* decreased (**Figures 6B, C**). *FvFUM1* encodes the key polyketide synthase located in the fumonisin biosynthetic cluster and is directly involved in fumonisin biosynthesis, whereas *FvZBD1* was traditionally not considered part of the cluster but in *F. verticillioides* is located directly adjacent and has been demonstrated to suppress fumonisin production when expressed at a high level (Proctor et al., 1999; Gao et al., 2020). This trend corresponded with decreasing amounts of aflatoxin remaining in the cultures, indicating that fumonisin production increased as aflatoxin was degraded either by *F. verticillioides* or by some other means (**Supplementary Table S3**). Like *A. flavus, F. verticillioides* also contains copies of both velvet regulators *veA* (*FvVE1*) and *laeA* (*FvLAE1*), whose expression was examined in this study (**Figures 6D, E**) (Calvo et al., 2016). At both 72 h and 96 h post inoculation, *FvVE1* expression was suppressed by aflatoxin in comparison to the control treatment. In the presence of aflatoxin, activation of *FvLAE1* was induced early at 72 h, whereas the control reached a similar level of expression only after 96 h.

## 4 Discussion

As recently reviewed by Chen et al. (2023), at least thirty studies over the last few decades across the globe have identified co-occurrences of both *A. flavus* and *F. verticillioides* in maize. Initially, these studies were relegated to warmer climates such as those in Africa, but as temperatures rise due to climate change, discovery of these fungal co-occurrences are spreading to traditionally more cooler climates. Several direct interaction studies under *in-vivo* and *in-vitro* conditions have been performed with the results varying greatly based on the experimental conditions (Camardo Leggieri et al., 2019; Chen et al., 2021; Lanubile et al., 2021). However, the reports overall agree that during competition each fungus influences the other’s production of their primary mycotoxin, but they never focused on the role the individual mycotoxins might have in these interactions.

The aim of this work was to determine the roles of mycotoxins of primary concern as regulators of competitive interactions between these two frequent commensal mycotoxigenic corn colonizing fungi. Here, we characterized the response to aflatoxin and fumonisin on its competitor’s growth and mycotoxin production. With null producing mutants, our results demonstrated that *F. verticillioides* inhibits the growth of *A. flavus* via fumonisin. Despite the inhibition caused by fumonisin, aflatoxigenic cultures of *A. flavus* still produce enough aflatoxin to inhibit fumonisin production in the *F. verticillioides* wild-type colony compared to the non-producing Δ*aflR* cultures. Fumonisin also inhibited aflatoxin accumulation in the reciprocal conditions, but this only occurred together with direct growth reduction. Consistent with the results of this report, work performed by Camardo Leggieri et al. (2019) and Chen et al. (2021) found that the presence of a competing fungus had significant effects on production of mycotoxins in *A. flavus* and *F. verticillioides* with coculture on plates and in planta showing reductions in both aflatoxin and fumonisin.

Exposing *A. flavus* spores to fumonisin B_1_ showed that, while toxic, the metabolite is not fungicidal. Even at a high dose of 100 µg/ml, while severely delayed, the spores eventually germinated followed by vegetative growth. Analysis of the fumonisin B_1_ content after adding it to liquid cultures of *A. flavus* demonstrated that fumonisin B_1_ concentration decreased by more than half that added (**Supplementary Table S3**). This decrease occurred by unknown means, but likely involved degradation by *A. flavus* itself. Regardless of how, this toxin decrease may explain the recovery seen with cultures exposed solely to fumonisin compared to *F. verticillioides* co-cultures where fumonisin continues to be actively produced, keeping *A. flavus* suppressed. This may also explain observations in the field, as it is likely to be more common that one species will establish itself on a host before a second can. With the initial establishment, the first species likely starts producing its secondary metabolites earlier; this allows it a competitive edge over other microbes. *F. verticillioides* was similarly antifungal and inhibitory towards *Fusarium graminearum* with fumonisin required for the effect (Sherif et al., 2023). The authors hypothesized that fumonisin accumulation in the seeds protects them from utilization by saprotrophic fungi giving a fitness advantage to fumonisin producing fungi. With *A. flavus* being a known opportunistic pathogen and saprotroph, the data here is consistent with the author’s hypothesis.

In terms of gene expression, fumonisin does not seem to have a direct effect on the aflatoxin biosynthetic gene cluster members. Even though expression of *aflM* was lower than the control at 96 h, this gene and *aflR* were unaffected at 72 h where aflatoxin production was already lower by nearly half in the treated sample compared to the control. In direct exposure to *F. verticillioides* or fumonisin, aflatoxin reduction only occurred at the proximal edge of interacting colonies or when exposed to high concentrations of fumonisin commensurate with visible growth inhibition (**Figure 2**). Thus, the effect of fumonisin treatment on key gene expression and on aflatoxin production appears to be a secondary effect of *A. flavus* growth inhibition.

In *A. flavus*, both VeA and LaeA are involved in secondary metabolism regulation and are noteworthy for their roles in regulation of aflatoxin biosynthesis by manipulating *aflR* (Amaike and Keller, 2009). Expression of both *veA* and *laeA* were suppressed in the fumonisin treatment. The interactions between proteins VeA, LaeA, and AflR are nuanced with imbalances in stoichiometry and self-regulating feedback loops impacting expression (Bok and Keller, 2004; Wang et al., 2022). Despite this, *laeA* expression was strongly suppressed when exposed to fumonisin B_1_.

Amaike and Keller (2009) speculated that the deletion of *laeA* in *A. flavus* resulted in defects in density based morphological development governed by an oxylipin quorum-like sensing system. Alterations in fungal quorum sensing through oxylipins may cascade into other defensive metabolic changes impacting fungal competition. Multiple oxylipins have been identified in both *Aspergillus* and *Fusarium* spp. that stimulate or inhibit production of mycotoxins (Liu et al., 2023). In the non-aflatoxigenic Δ*aflR* strain, suppression of fumonisin production by *F. verticillioides* was detected, albeit more weakly. This suggests that other *A. flavus* secondary metabolites, beyond aflatoxin, are produced that combat *F. verticillioides.* These other compounds may also be activated by changes in the expression of global regulators like VeA and LaeA.

For humans and animals, due to the high toxicity of consumed aflatoxin, regulations from the U.S. Food and Drug Administration place content limits in the part per billion (ppb) range for food (20 ppb) and feed (100 ppb). Under aflatoxin exposure up to 100 ppm, *F. verticillioides* appeared resistant to visible effects on colony growth. Interestingly, fumonisin production was severely inhibited by aflatoxin producing strains of *A. flavus* in a colony-wide response.

When *F. verticillioides* was exposed to 20 µg/ml of AFB_1_, fumonisin was still produced and found at higher levels than in the solvent control treatment. Expression of *FvFum1* increased by more than 50% from 72 h to 96 h in the dosed samples. Intriguingly, this corresponded to a reduction of more than 50% in *FvZBD1* expression over the same time frame. In a previous study, the gene *FvZBD1* was identified as a highly induced negative regulator of fumonisin production in *F. verticillioides* (Gao et al., 2020). In that work, the xenobiotic compound pyrrocidine, produced by the corn kernel colonizing fungus and potential biological control agent, *Sarocladium zeae*, induced *FvZBD1* 4000-fold, coincident with dramatic suppression of fumonisin production in exposed *F. verticillioides*. Further deletion of *FvZBD1* resulted in high fumonisin accumulation, consistent with its role as a suppressor. It appears that, like pyrrocidine, aflatoxin acts in a similar way suppressing fumonisin production via *FvZBD1* induction albeit at a reduced capacity. Analysis of aflatoxin in those samples revealed that the total amount of AFB_1_ was reduced by 24% at 72 h and 37% at 96 h (**Supplementary Table S3**), suggesting *F. verticilloides* is degrading or biotransforming aflatoxin to better survive. This tolerance was also seen in solid agar where the amount of fumonisin produced in response to an aflatoxin concentration of 50 µg/ml was not significantly different than a dose twice as much at 100 µg/ml (**Figure 4**). Strangely though when *F. verticillioides* is directly exposed to an aflatoxin producing *A. flavus* the suppression of fumonisin (**Figure 2**) is much greater than when it is exposed to a high direct dose of aflatoxin (100 µg/ml). This is surprising because based on LC/MS data the *A. flavus* colony is only producing aflatoxins at levels around or below 100 ng/ml. This would indicate that *F. verticillioides* can handle high acute exposures of aflatoxin but suffers from chronic exposure such as when growing near a producing colony. It is worth noting that this effect may be due to additional secondary metabolites produced by *A. flavus* which may have a synergistic effect with aflatoxin on *F. verticillioides.*

Like *Aspergillus, Fusarium* spp. have functional homologs of the velvet complex including *veA* (*FvVE1)* and *laeA* (*FvLAE1). FvVE1* and *FvLAE1* are both positive regulators of fumonisin production, and as with *A. flavus*, the expression of these genes were also examined [ (Butchko et al., 2012) (Myung et al., 2012)]. Expression of *FvVE1* was lower at both 72 h and 96 h in aflatoxin treated samples compared to the control. On the other hand, *FvLAE1* in the treated sample had an earlier activation at 72 h. Suppression of *FvVE1*, while significant, does not appear to be repressed to the extent *FvZBD1* is. Butchko et al. (2012) presented microarray data that demonstrated that *FvZBD1* is positively regulated by *FvLAE1.* The interaction between *FvZBD1* and the velvet complex, including *FvLAE1*, has not been thoroughly investigated, it is possible genetic regulation involving the velvet complex plays a role in both organisms’ response to the other.

## 5 Conclusion

*A. flavus* and *F. verticillioides* are known to interact while colonizing corn seed, but there has been a lack of knowledge regarding the specifics of this interaction. Here, we focused on the role aflatoxin and fumonisin play when *A. flavus* and *F. verticillioides* directly compete. Our results indicate that fumonisin suppresses the growth of *A. flavus* and the expression of the global secondary metabolism regulator *laeA*. This is an interesting result as it appears when *A. flavus* encounters *F. verticillioides* one of its responses is to start secreting more aflatoxin. This response does appear to be defensive in nature since aflatoxin induces expression of the fumonisin repressing gene *FvZBD1*. Our results indicate that both aflatoxin and fumonisin had an effect on the expression of the velvet complex in both competing fungi. The velvet complex is known for its global role in regulating fungal secondary metabolism. This suggests that fumonisin and aflatoxin are just the opening salvos when these fungi battle for the same resources or host.

## Data Availability

All relevant data is presented in the publication. Data requiring upload to a third party (like sequencing data) was not used in this study.
