# Supplementary material for: Fungal chemical warfare: the role of aflatoxin and fumonisin in governing the interaction between the maize pathogens, Aspergillus flavus and Fusarium verticillioides
Source: Front Cell Infect Microbiol. 2025 Jan 3;14:1513134. doi: 10.3389/fcimb.2024.1513134 (PMC11739330; doi:10.3389/fcimb.2024.1513134)
Supplement: Supplementary file 1 [file Table1.docx]

**
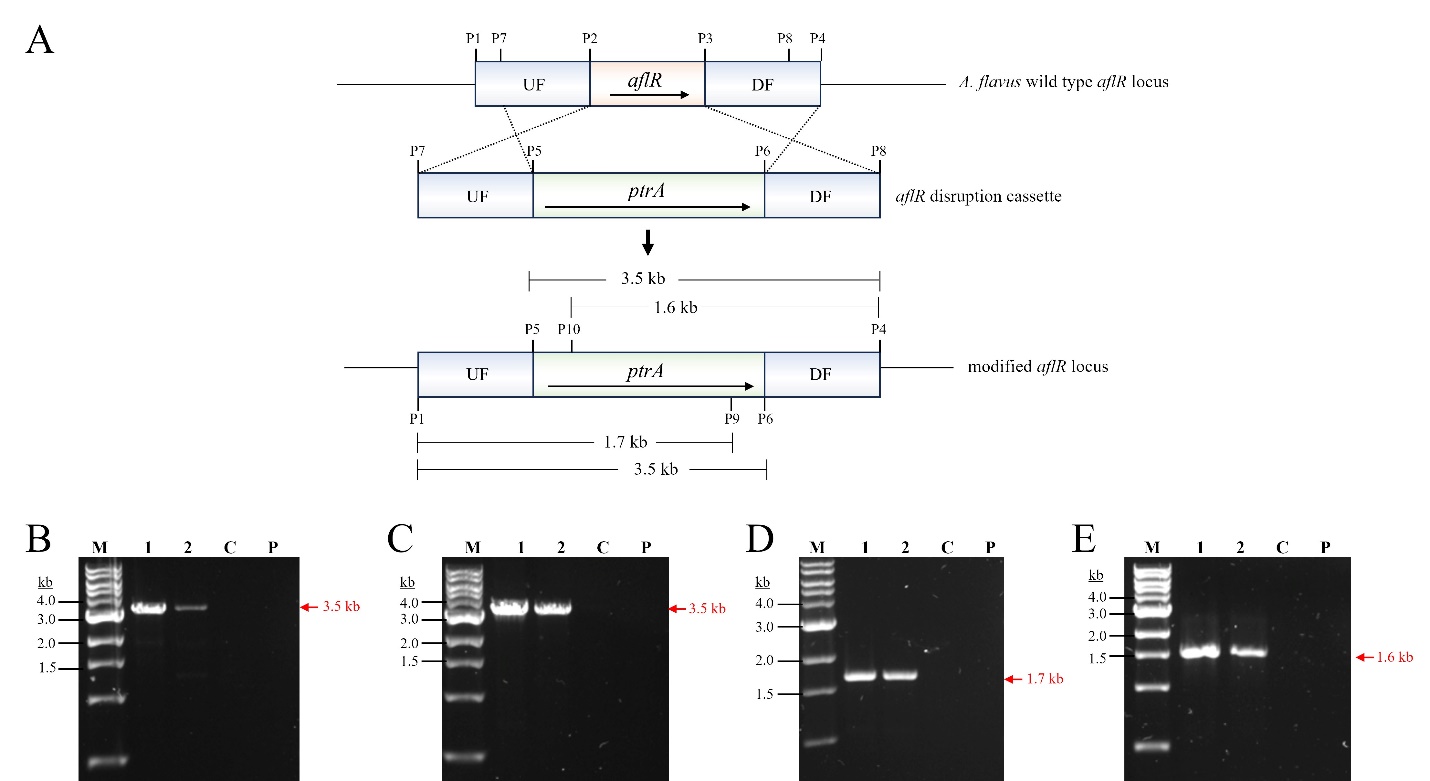
**

**Figure S1: Construction and confirmation of an *Aspergillus flavus* Δ*aflR* strain**. (a) Schematic diagram representing the replacement of the *aflR* coding region with the *pyrithimaine resistance* gene (*ptrA*) by a double homologous recombination event. Abbreviations UF: Upstream *aflR* flanking sequence; DF: Downstream *aflR* flanking sequence. Successful replacement of the *aflR* coding region with ptrA was confirmed with four separate diagnostic PCR’s using the primers (b) P1/P6, (c) P4/P5, (d) P1/P9, and (e)P4/10 to generate PCR product sizes of 3.5 kb, 3.5 kb, 1.7 kb, and 1.6 kb in the *alfR* mutant strain. Since one primer bound in the *ptrA* gene for each PCR conducted, no PCR band was expected in the control PCRs. The Δ*aflR* 1 strain was selected for further assays utilized in this study and denoted as Δ*aflR* in the text. Abbreviations **M**: Marker (New England Biolabs, Catalog number: N0552); **1**: Δ*aflR* 1; **2**: Δ*aflR* 2; **C**: NRRL3357 Wild-type, **P**: pPTRI vector DNA.

**Table S1** Primers used in Strain Construction.

| Primer name | Sequence (5’ to 3’) |
| --- | --- |
| P1: aflRF1-F | CCAGTTCATCCGAGTGAGC |
| P2: aflRF1-R | GATCCCGTAATCAATTGCCCTGGAGGTGAGGAAGGAATTC |
| P3: aflRF2-F | AAGAGCGGCTCATCGTCAAGATGACAGGCCGGTTTC |
| P4: aflRF2-R | CCCATACCATTCGACCATG |
| P5: ptrI-F | GGGCAATTGATTACGGGATC |
| P6: ptrI-R | TGACGATGAGCCGCTCTT |
| P7: aflR nest-F | AGCTGCCGCTTAACCTTGG |
| P8: aflR nest-R | GCCTTTCTCCGTCGATAAGTC |
| P9: ptrI175-R | CCGTATAGATCAGCGGCAC |
| P10: ptrI1921-F | GTGTCCCGTATGTAACGGTG |

**Table S2** Primers used in qRT-PCR Analysis.

| Species | Primer | Sequence |
| --- | --- | --- |
| *A.flavus* | *18S* | F - TTCCTAGCGAGCCCAACCT |
|  |  | R - CCCGCCGAAGCAACTAAG |
|  | *aflR* | F - GTATGATGGGCGAGGATTG |
|  |  | R - CTAAACCCGAGTAGTGAGAAAG |
|  | *aflM* | F - GAATTTGACCGGGTCTTCC |
|  |  | R - CACGGTGATCTTCTTGTCTC |
|  | *veA* | F - CGTTCCGTTCACCGTATTT |
|  |  | R - GTAGACTCTCTCCTCATCGTAG |
|  | *laeA* | F - GCGCCTTTCAGTCGTATT |
|  |  | R - GTAGGCGTGTATTTCTTTGTTG |
| *F. verticillioides* | *FvTUB2* | F - CAGCGTTCCTGAGTTGACCCAACAG |
|  |  | R - CTGGACGTTGCGCATCTGATCCTCG |
|  | *FvFUM1* | F- GCGATATTGATCCGCAGCAG |
|  |  | R - TCTCCCCATACGCCAACGTA |
|  | *FvZBD1* | F - GGTCTCAGGAGGATTGCTAAAG |
|  |  | R - GCACGATACTGAACCAGAGATAG |
|  | *FvVE1* | F - TCCTACTTCTCAGTCTCCATAC |
|  |  | R - GTAGATTGTCTGCGGTCATAC |
|  | *FvLAE1* | F -GGAGGTGATCAAGGCTTATG |
|  |  | R - GAGACAGCCCGATATTGAAC |

**Table S3** Reduction of Competitor’s Mycotoxin at 72 and 96 h.

| Experiment | 72 h | 96 h |
| --- | --- | --- |
| *A. flavus* dosed with FB_1_ | Fumonisin B_1_ (µg/ml) | |
|  | 7.110 +/- 0.141 | 6.359 +/ -0.027 |
| *F. verticillioides* dosed with AFB_1_ | Aflatoxin B_1_ (µg/ml) | |
|  | 15.195 +/- 0.210 | 12.7195 +/- 0.273 |
